# FABP1 and FABP2 as markers of diabetic nephropathy

**DOI:** 10.7150/ijms.49078

**Published:** 2020-08-27

**Authors:** I-Ting Tsai, Cheng-Ching Wu, Wei-Chin Hung, Thung-Lip Lee, Chin-Feng Hsuan, Ching-Ting Wei, Yung-Chuan Lu, Teng-Hung Yu, Fu-Mei Chung, Yau-Jiunn Lee, Chao-Ping Wang

**Affiliations:** 1 Department of Emergency, E-Da Hospital, Kaohsiung 82445 Taiwan.; 2 Division of Cardiology, E-Da Hospital, Kaohsiung 82445 Taiwan.; 3 Division of Endocrinology and Metabolism, Department of Internal Medicine, E-Da Hospital, Kaohsiung 82445 Taiwan.; 4 Division of General Surgery, Department of Surgery, E-Da Hospital, Kaohsiung 82445 Taiwan.; 5 School of Medicine, College of Medicine, I-Shou University, Kaohsiung 82445 Taiwan.; 6 The School of Chinese Medicine for Post Baccalaureate, College of Medicine, I-Shou University, Kaohsiung 82445 Taiwan.; 7 School of Medicine for International Students, College of Medicine, I-Shou University, Kaohsiung 82445 Taiwan.; 8 Lee's Endocrinologic Clinic, Pingtung 90000 Taiwan.; 9 Division of Cardiology, Department of Internal Medicine, E-Da Cancer Hospital, Kaohsiung 82445 Taiwan.

**Keywords:** Type 2 diabetes mellitus, diabetic nephropathy, fatty acid-binding protein 1, fatty acid-binding protein 2

## Abstract

**Background:** Diabetes mellitus is the leading cause of diabetic nephropathy and a major public health issue worldwide. Approximately 20-30% of patients with type 2 diabetes mellitus (T2DM) have renal impairment. Fatty acid-binding protein 1 (FABP1) is expressed in renal proximal tubule cells and released into urine in response to hypoxia caused by decreased peritubular capillary blood flow, and FABP2 is responsible for the transport of free fatty acids in the intestinal endothelium cells. There is increasing evidence that FABP1 and FABP 2 play a role in the development and progression of chronic kidney disease. The aim of this study was to investigate the relation of circulating FABP1 and FABP2 levels to nephropathy in patients with T2DM.

**Methods:** For this study, 268 subjects with T2DM who were enrolled in a disease management program were stratified according to urinary microalbumin and serum creatinine measurements. The plasma FABP1 and FABP2 concentrations were examined by enzyme-linked immunosorbent assay. Demographic and potential metabolic confounding factors were analyzed with logistic regression to calculate the effects of FABP1 and FABP2 levels on diabetic nephropathy.

**Results:** The FABP1 and FABP2 levels increased in parallel with the advancement of diabetic nephropathy. Increasing concentrations of FABP1 and FABP2 were independently and significantly associated with diabetic nephropathy. Multiple logistic regression analysis revealed FABP1 and FABP2 as an independent association factor for diabetic nephropathy, even after full adjustment of known biomarkers. Furthermore, receiver operating characteristic curve analysis showed that a FABP1 level of >33.8 ng/mL and a FABP2 level of >2.8 ng/mL were associated with diabetic nephropathy.

**Conclusion:** Our results suggest that FABP1 and FABP2 may be novel biomarkers of diabetic nephropathy.

## Introduction

Diabetic nephropathy is common. One in 4 women and one in 5 men with type 2 diabetes mellitus (T2DM) develops diabetic nephropathy. It is even more common in type 1 diabetes. Diabetic nephropathy is a syndrome characterized by the presence of pathological quantities of urine albumin excretion, diabetic glomerular lesions, and loss of glomerular filtration rate (GFR) in diabetics. The diabetes epidemic has resulted in diabetic nephropathy becoming the most frequent cause of end-stage renal disease (ESRD) in most countries. Therefore, early diagnostic markers for monitoring and predicting the progression of diabetic nephropathy are needed to enable the timely administration of the most appropriate protective treatments.

Tubulointerstitial injury has been suggested to have an important impact on the progression of diabetic nephropathy 1. Fatty acid-binding protein 1 (FABP1) (also known as liver-type fatty acid-binding protein or L-FABP) is a 14 kDa small molecule that is expressed in the proximal tubules of the human kidney and participates in fatty acid metabolism 2. The circulating fraction of FABP1 is filtered by the glomeruli and afterwards reabsorbed in the proximal renal tubules, which explains the increase of its concentration in the urine when proximal tubule cell injury occurs 3. Previous animal studies of kidney disease have reported an upregulated expression of the human FABP1 gene in the kidneys, and that stress can cause increases in the urinary excretion of human FABP1 (e.g., urinary protein overload 4, tubular stretch 5, tubular ischemia 6, toxins 7, hyperglycemia 8, and hypertension 9). In addition, a clinical study reported associations between the urinary excretion of FABP1 and the severity of tubulointerstitial damage and rate of chronic kidney disease (CKD) progression in patients with non-diabetic CKD 10. These findings suggest that urinary FABP1 may be a clinical marker to screen for kidney dysfunction and identify patients who are likely to experience deterioration in renal function in the future.

Fatty acid-binding protein 2 (FABP2) (also known as intestinal-type fatty acid-binding protein or I-FABP) is a low molecular weight (14-15 kDa) cytosolic, water-soluble protein specifically expressed by enterocytes from the duodenum to the ileum 11. FABP2 is rapidly released into the systemic circulation on enterocyte injury, and accordingly has been shown to be a useful biomarker for diagnosing acute intestinal ischemia, including necrotizing enterocolitis 12 and nonocclusive mesenteric ischemia 2,4. FABP2 is thought to be rapidly cleared by the kidneys (half-life of approximately 11 minutes) similar to other members of the FABP multigene family 13. Although it can be removed by renal replacement therapy, FABP2 levels in patients with renal insufficiency are usually elevated. In addition, a previous study found that FABP2 levels in nondiabetic patients with CKD and pre- hemodialysis (HD) ESRD were significantly higher than those in patients with normal renal function, and suggested that it could be used as a diagnostic and prognostic marker in patients with renal insufficiency 14.

The purpose of the present study was to investigate the levels of FABP1 and FABP2 in T2DM patients in various stages of nephropathy to clarify the role of FABP1 and FABP2 in the pathogenesis of diabetic nephropathy. The relationship between the 2 selected markers and clinical and biochemical parameters were also evaluated.

## Methods

### Participants

From January 2017 to December 2018, 268 consecutive patients with diabetes who visited the diabetic or cardiovascular clinics at E-Da Hospital were enrolled. The diagnosis of T2DM was based on the World Health Organization criteria 15. Patients presenting with symptoms suggestive of type 1 diabetes, including diabetic ketoacidosis, acute presentation with heavy ketonuria (3+), or continuous requirement for insulin within 1 year of diagnosis were excluded 16. Patients with a urinary tract infection, urolithiasis, liver cirrhosis, congestive heart failure, chronic lung diseases, chronic otitis media, sinusitis, chronic viral hepatitis, pelvic infection, and other known renal diseases were also excluded on the basis of interviews, physical examinations, and urinalysis. The mean age of the subjects was 67.1±9.8 years, and 69.4% were female. This study was approved by the Human Research Ethics Committee of Kaohsiung E-Da Hospital. Written informed consent was obtained from each participant before enrolment.

### Data collection

Each patient received a detailed interview about his or her personal disease history and smoking history. Information on smoking habits was assessed using a standardized questionnaire. The patients' smoking status was classified as never having smoked, former smoking (ceased smoking for at least 1 year), or current smoking. In this study, former and current smokers were analyzed as a group and compared with those who had never smoked. All of the study subjects were of Han Chinese origin, without any known ancestors of other ethnic origin, and living in the same region at the time of the study. All patients underwent a complete physical examination and routine blood and urine biochemical analyses, and were assessed for the presence and extent of macrovascular or microvascular diabetic complications. Waist and hip circumferences were measured to the nearest 0.1 cm at the narrowest point between the lowest rib and the uppermost lateral border of the right iliac crest, and the hips were measured at their widest point. The body mass index (BMI) and waist to hip ratio (WHR) were calculated for each subject. A trained nurse measured blood pressure (BP) with a digital automatic BP monitor (Omron, model HEM-907, Omron, Japan) after the subjects had rested for 5 minutes. In addition, venous blood was drawn in the morning after an overnight fast. Serum creatinine was analysed according to the kinetic Jaffé method on a SYNCHRON CX System analyzer (SYNCHRON, Los Angeles, CA) using reagents from Beckman (Beckman Coulter Diagnostic, Los Angeles, CA). Serum triglycerides (TGs), total cholesterol, low-density lipoprotein cholesterol (LDL-C), high-density lipoprotein cholesterol (HDL-C), albumin, hemoglobin, glucose, and white blood cell (WBC) count were determined using standard commercial methods on a parallel-multichannel analyzer (SYNCHRON, Los Angeles, CA). Hemoglobin A1c (HbA1c) was measured using high performance liquid chromatography.

Diabetic patients were screened based on the results of the urinary analysis and urinary microalbumin and serum creatinine measurements. They were classified as having normal albuminuria (urinary albumin-to-creatinine ratio [UACR] <30 mg/g), microalbuminuria (UACR 30-300 mg/g, with at least two or more tests showing significant results), or overt nephropathy (UACR >300 mg/g and/or serum creatinine >1.5 mg/dl). Estimated glomerular filtration rates (eGFRs) were calculated using the CKD-EPI two-concentration race equation 17. The fatty liver index (FLI) was calculated according to a previously published report by Bedogni et al. 18: FLI = [e^0.953^×log_e_ (TGs) + 0.139×BMI + 0.718×log_e_ (gamma-glutamyl- transferase, GGT) + 0.053×waist circumference-15.745)] / [1+ e^0.953^×log_e_ (TGs) + 0.139×BMI + 0.718×log_e_ (GGT) + 0.053×waist circumference-15.745] ×100, with TGs measured in mmol/l, GGT in U/l, and waist circumference in cm.

### Plasma FABP1 and FABP2 measurements

All blood samples were drawn after overnight fasting, and plasma samples were kept at -80°C for subsequent assay. The concentrations of plasma FABP1 and FABP2 were determined using commercial enzyme-linked immunosorbent assay (ELISA) kits (Cloud-Clone Corp., Katy, USA and R&D Systems, Inc., Minneapolis, USA). The analytical sensitivities were 0.59 ng/mL for FABP1 and 3.63 pg/mL for FABP2. ELISA was performed as per the instructions of the manufacturer. According to the manufacturer, the FABP1 and FABP2 ELISA had excellent specificity for the detection of human FABP1 and FABP2, and no significant cross-reactivity or interference with analogues was observed. Samples were measured in duplicate in a single experiment.

### Statistical analysis

Data normality was analyzed using the Kolmogorov-Smirnov test. Continuous, normally distributed variables are presented as mean±SD, and non-normally distributed variables as median (interquartile range). Statistical differences in variables were compared using a one-way ANOVA for variables of normal distribution followed by the Tukey pairwise comparison. Categorical variables are presented as frequencies and/or percentages, and inter-group comparisons were analyzed using the chi-square test. Since the distributions of serum TGs, plasma FABP1 and FABP2 were skewed, logarithmically transformed values were used for the statistical analysis.

Associations between FABP1 and FABP2 with diabetic nephropathy were examined using multivariate logistic regression analysis that contained: (1) FABP1 or FABP2, age and gender, (2) FABP1 or FABP2, age, gender, BMI, systolic blood pressure (SBP), and diastolic blood pressure (DBP), (3) FABP1 or FABP2, age, gender, BMI, SBP, DBP, and fasting glucose, and (4) FABP1 or FABP2, age, gender, BMI, SBP, DBP, fasting glucose, lipid profile, and smoking status. We further divided the distribution of FABP1 or FABP2 in pooled data into tertiles, and used general linear and logistic regression models to estimate significant trends across increasing tertiles and to estimate the odds ratio (OR) of diabetic nephropathy in each tertile using the lowest tertile as a reference category. Multivariate-adjusted ORs are presented with 95% confidence intervals (CIs). Pearson's correlation coefficients and simple linear regression analysis were used to examine the correlations and independence among plasma FABP1 and FABP2 and the values of other parameters. Receiver operating characteristic (ROC) curves were used to obtain the specificity and sensitivity of plasma FABP1 and FABP2 to distinguish patients with diabetic nephropathy from those without diabetic nephropathy. Statistical significance was accepted if p <0.05. All statistical analyses were performed using SAS statistical software, version 8.2 (SAS Institute Inc., Cary, NC).

## Results

### Clinical characteristics of study subjects

A total of 268 type 2 diabetic patients were included in this cross-sectional study. The clinical and biochemical characteristics of the patients stratified by nephropathy status are given in Tables 1 and 2. The prevalence of normoalbuminuria, microalbuminuria, and overt nephropathy in the present study was 64.9, 25.0, and 10.1%, respectively. Patients with nephronpathy were younger and higher SBP, DBP, and FLI than those without albuminuria. Furthermore, patients with overt nephropathy had a higher prevalence of hypertension, smokers, insulin alone, oral hypoglycemic agent/insulin, and angiotensin II receptor blocker/angiotensin-converting enzyme inhibitor treatment, and lower frequency of oral hypoglycemic agent treatment than those without albuminuria (**Table 1**).

### Biochemical characteristics of study subjects

There was a significant differences in FABP1 and FABP2 levels (*p* <0.01) across the three study groups, with the main difference being detected between overt nephropathic and normoalbuminuric subjects. The mean FABP1 and FABP2 levels increased parallel to the severity of nephropathy (**Table 2**). Furthermore, the patients with overt nephropathy had higher HbA1c, total cholesterol, LDL-C, and creatinine levels, and lower albumin concentrations than those without albuminuria. In addition, the patients with overt nephropathy also had higher uric acid and UACR, and lower eGFR, red blood cell (RBC) count, and hemoglobin than the without albuminuria and microalbuminuria groups (**Table 2**).

### Associations between plasma FABP1 and FABP2 and diabetic nephropathy

The plasma FABP1 and FABP2 concentrations were significantly associated with diabetic nephropathy even after controlling for anthropometric variables, fasting glucose, lipid profile, and smoking status (**Table 3**). Increasing levels of FABP1 and FABP2 showed a significant linear trend and were independently associated with diabetic nephropathy, especially when concentrations were analyzed both by tertile and by a continuous variable (**Tables 3 and 4**). In the multiple logistic regression analysis, the fully adjusted ORs for diabetic nephropathy in the second and third tertiles of FABP1 were 3.47 (95% CI: 1.59-7.88) and 4.22 (95% CI: 1.85-10.03), respectively, and the fully adjusted ORs for diabetic nephropathy in the second and third tertiles of FABP2 were 1.34 (95% CI: 0.65-2.79) and 1.62 (95% CI: 1.50-11.81), respectively (**Table 4**).

### Correlations among FABP1 and FABP2 and clinical and biochemical parameters

**Figure 1:**
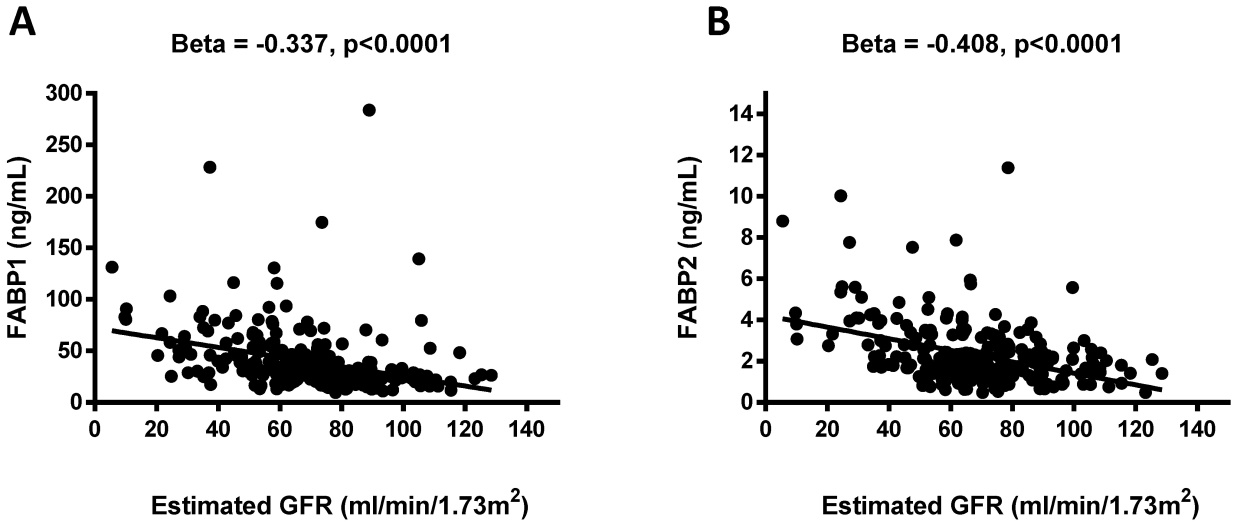
Association between plasma concentrations of fatty acid-binding protein 1 (FABP1) and FABP2 and estimated glomerular filtration rate (eGFR) (**A and B**). Plasma FABP1 and FABP2 concentrations were significantly and negatively associated with eGFR.

Negative associations were observed between the FABP1 and FABP2 levels and eGFR (β = -0.337, *p* <0.0001 and β = -0.408, *p* <0.0001; **Figure 1**). Pearson's correlation analysis revealed that FABP1 level was positively correlated with age, BMI, WHR, uric acid, creatinine, FABP2, and FLI, and negatively correlated with eGFR, albumin, RBC, and hemoglobin. In addition, FABP2 level was positively correlated with SBP, serum total cholesterol, uric acid, creatinine, FABP1, and FLI, and negatively correlated with eGFR, albumin, RBC, and hemoglobin (**Table 5**).

### FABP1, FABP2, and diabetic nephropathy

The ROC curve to detect diabetic nephropathy revealed an area under the curve (AUC) of 0.780 (95% CI: 1.019-1.045, *p* <0.0001) for FABP1. A plasma FABP1 concentration of >33.8 ng/mL was associated with diabetic nephropathy, with a sensitivity of 75.3% and specificity of 75.6%. In addition, the ROC curve to detect of diabetic nephropathy revealed an AUC of 0.690 (95% CI: 1.302-1.976, *p* <0.0001) for FABP2. A plasma FABP2 concentration of >2.8 ng/mL was associated with diabetic nephropathy, with a sensitivity of 48.2% and specificity of 85.6% (data not shown).

## Discussion

In the present study, we demonstrated that plasma FABP1 levels were positively correlated with age, BMI, WHR, uric acid, creatinine, FABP2, and FLI, and negatively correlated with eGFR, albumin, RBC, and hemoglobin. In addition, we demonstrated that plasma FABP2 level was positively correlated with SBP, serum total cholesterol, uric acid, creatinine, FABP1, and FLI, and negatively correlated with eGFR, albumin, RBC, and hemoglobin. Moreover, an increased concentration of plasma FABP1 and FABP2 was associated with diabetic nephropathy, even in a fully adjusted model. Furthermore, the ROC curves of FABP1 and FABP2 concentrations showed that a FABP1 concentration of >33.8 ng/mL was associated with diabetic nephropathy and a FABP2 concentration of >2.8 ng/mL were associated with diabetic nephropathy. To the best of our knowledge, this is the first study to investigate the relationship between FABP1 and FABP2 and diabetic nephropathy in patients with T2DM.

Diabetic nephropathy is a major problem causing increased morbidity and mortality as the increase in total number of diabetic patients finds a reflection in increased prevalence of diabetic patients in ESRD population, and therefore identifying patients with diabetic nephropathy early is of great importance both to allow for timely interventions and to improve prognoses. Previous studies have shown that the concentration of FABP1 in urine can serve as a useful marker for the diagnosis of early-stage kidney damage, and especially acute kidney injury 19, 20. FABP1 is a protein found in the cytoplasm of both healthy and injured proximal tubule cells in the kidneys 21. Various pathological conditions including hyperglycemia, hypertension, proteinuria, and toxin-induced injury to the proximal tubule cells may result (either through the regulation of gene expression or directly) in an increase in the excretion of urine-derived FABP1 21,22. Previous studies have also demonstrated that FABP1 can play an important role in injury and repair processes in the kidneys, and that the monitoring of urine FABP1 concentration may make it possible to predict the occurrence and severity of various renal diseases 19,23. In the present study FABP1 levels were associated with diabetic nephropathy, and plasma FABP1 levels increased in parallel with the decline in eGFR (β = -0.337, *p* <0.0001) (**Figure 1**). Several previous studies have reported a strong correlation between urinary FABP1 levels and eGFR in patients with T2DM 24,25. Suzuki et al. reported that urinary FABP1 levels were significantly higher in patients with macroalbuminuria than in those with microalbuminuria 24. It is also well known that the tubular system plays an important role in the pathophysiology of diabetic nephropathy. CKD patients have massive proteinuria and fatty acids overload in proximal tubules, and hypertriglyceridemia may also cause fatty acid overload, as well 26. In addition, the urinary excretion of arachidonic acids and linoleic has been found to be significantly higher in CKD patients than in patients with minimal change nephrotic syndrome 27,28. Nonoxidized fatty acids appear to be cytotoxic after peroxidation, and may also provoke macrophage infiltration, the production of inflammatory factors, and accelerate the progression of tubulointerstitial damage 7,9,28. This may explain why our diabetic nephropathy patients had high plasma FABP1 levels.

In terms of CKD, animal and clinical studies have documented the usefulness of FABP1 as a marker for diabetic kidney disease. Kamijo-Ikemori et al. reported a significant increase in the expression of FABP1 in diabetic mice compared to control mice 7. In addition, Panduru et al. demonstrated that FABP1 was an indicator of the development of diabetic kidney disease in humans, regardless of its stage 29. Mou et al. showed a correlation between the level of FABP1 in urine and the development of renal impairment in chronic glomerulonephritis patients, and suggested that FABP1 excreted in the urine may be a good marker of the progression of chronic glomerulonephritis 30. Moreover, the level of FABP1 in urine has been demonstrated to be a novel biomarker for renal damage, and a rapid kit to measure the concentration of FABP1 in urine has been introduced for clinical use in Japan 31. The results of the present study support the idea 29-31 that FABP1 may be a marker of diabetic nephropathy in patients with T2DM.

The second marker investigated in our study was FABP2, which has been proposed to be a possible non-invasive marker to evaluate gut wall integrity loss and inflammation 32. We found high plasma levels of FABP2 in our patients with diabetic nephropathy. Most patients with critical illnesses also have renal problems 33, and these problems likely alter FABP2 levels. Recently, FABP2 has been shown to be a reliable marker of acute intestinal ischemia, including nonocclusive mesenteric ischemia 13, for which renal failure and hemodialysis are well known major risk factors 34,35. However, the influence of renal function on the level of FABP2 has not been elucidated in previous studies that investigated the diagnostic utility of FABP2 13,36,37.

Our results showed that plasma FABP2 levels were positively correlated with SBP, serum total cholesterol, uric acid, creatinine, FABP1, and FLI, and negatively correlated with eGFR, albumin, RBC, and hemoglobin. This suggests that the concentration of plasma FABP2 increases with the progression of kidney disease. Okada et al. reported that plasma FABP2 levels in patients with CKD and pre-HD ESKD patients were significantly higher than those in patients with a normal renal function. Furthermore, they found that post-HD FABP2 levels in the patients with ESKD were significantly decreased, nearly to the level in patients with normal renal function 14. Accordingly, clinicians and researchers should consider using FABP2 as a diagnostic and prognostic marker in patients with renal insufficiency. Although the use of plasma FABP2 level as a single surrogate biomarker to predict diabetic nephropathy will be limited, it may be useful as one indicator in a multi-marker panel such as in combination with plasma FABP1 to better assess individuals suspected of having diabetic nephropathy.

There are several limitations to this study. The cross-sectional design limits our ability to infer a causal relationship between increased plasma FABP1 and FABP2 levels and diabetic nephropathy. Studies with long-term follow-up are needed to clarify the role of FABP1 and FABP2 in association with diabetic nephropathy. Furthermore, the number of enrolled patients was relatively small. However, this is a cross-sectional study, and cross-sectional studies are the best way to determine prevalence but do not allow for robust comparisons. Moreover, this study only included patients with diabetic nephropathy, and therefore our results may not be generalizable to the general population. Additional studies that include a larger multi-ethnic cohort are needed to investigate these associations. In addition, it is important to study this prognostic value in diabetic patients with increasing FABP levels who do not yet have microalbuminuria, or GFR loss. Prospective cohort observation is undergoing to test the prognostic value of circulating FABP in the prediction of development or progression of diabetic nephropathy. Finally, whether increased FABP2 levels are associated with diabetic nephropathy requires further research.

## Conclusions

In conclusion, this study demonstrated that plasma FABP1 and FABP2 levels were significantly associated with diabetic nephropathy. FABP1 and FABP2 could be novel biomarkers of diabetic nephropathy.

## Figures and Tables

**Table 1:** Clinical characteristics of study subjects

Parameter	Normoalbuminuria	Microalbuminuria	Overt nephropathy	*p* value
N	174	67	27	
Age (years)	63.3±9.5	66.8±9.6	62.1±11.1	0.025^#^
Gender, female (n, %)	123 (70.7)	47 (70.2)	16 (59.3)	0.482*
Hypertension (n, %)	91 (52.3)	55 (82.1)	24 (88.9)	<0.0001*
Hyperlipidemia (n, %)	126 (72.4)	50 (74.6)	22 (81.5)	0.600*
Smokers (n, %)	18 (10.3)	13 (19.4)	7 (25.9)	0.036*
**Medications** **(n, %)**				
Oral hypoglycemic agent	171 (98.3)	62 (92.5)	19 (70.4)	<0.0001*
Insulin alone	28 (16.1)	21 (31.3)	19 (70.4)	<0.0001*
Oral hypoglycemic agent + insulin	26 (14.9)	16 (23.9)	11 (40.7)	0.005*
ARB and ACEi use	73 (42.0)	50 (74.6)	24 (88.9)	<0.0001*
Statin use	120 (69.0)	49 (73.1)	21 (77.8)	0.578*
Duration of diabetes (years)	14.6±7.4	15.4±7.8	16.9±8.1	0.335^#^
Body mass index (kg/m^2^)	25.7±4.4	26.3±5.5	27.9±4.1	0.064^#^
Waist-to-hip ratio	0.92±0.08	0.94±0.08	0.94±0.08	0.121^#^
Systolic blood pressure (mmHg)	132±17	142±17	150±14	<0.0001^#^
Diastolic blood pressure (mmHg)	73±10	78±9	83±11	<0.0001^#^
Fatty liver index	1.8±0.8	2.2±0.8	2.4±0.8	0.0001^#^

Data are mean ± SD, frequency (percent), or median (interquartile range). ARB, angiotensin II receptor blocker; ACEi, angiotensin-converting enzyme inhibitor. *p values were calculated by Chi-square test for categorical data. ^#^*p* values were calculated by one-way ANOVA test followed by the Tukey pairwise comparison for numerical data.

**Table 2:** Biochemical characteristics of study subjects

Parameter	Normoalbuminuria	Microalbuminuria	Overt nephropathy	*p* value^#^
N	174	67	27	
Fasting glucose (mg/dl)	139.8±37.1	148.2±48.6	143.7±42.6	0.353
HbA1c (%)	7.2±1.2	7.6±1.3	7.7±1.4	0.028
Total cholesterol (mg/dl)	172.3±27.8	177.8±28.5	192.7±63.0	0.010
Triglycerides (mg/dl)	88.0 (63.0-125.3)	116.0 (79.0-153.0)	111.0 (82.0-182.0)	0.076
HDL cholesterol (mg/dl)	58.9±16.6	53.8±12.7	55.2±14.8	0.057
LDL cholesterol (mg/dl)	84.4±22.9	93.4±24.6	102.8±52.5	0.002
Uric acid (mg/dl)	5.0±1.8	5.6±1.8	6.6±1.5	<0.0001
Creatinine (mg/dl)	0.9±0.2	1.0±0.3	1.6±0.9	<0.0001
Estimated GFR (ml/min/1.73m^2^)	82.8±19.9	72.7±18.9	52.7±27.3	<0.0001
UACR (mg/g)	5.1 (1.6-13.4)	63.3 (42.9-97.7)	799.4 (432.8-1114.6)	<0.0001
Hemoglobin (g/dl)	13.3±1.5	12.9±1.8	11.9±1.9	0.0003
Albumin (g/dl)	4.4±0.3	4.4±0.3	4.2±0.3	0.0013
White blood cell (10^9^/l)	6665±1700	7609±2079	7316±2057	0.0012
Red blood cell (× 10^6^/μl)	467±58	470±78	424±87	0.0054
Fatty acid-binding protein 1 (ng/ml)	27.6 (20.8-39.6)	32.6 (26.2-46.6)	50.5 (31.1-80.7)	0.001
Fatty acid-binding protein 2 (ng/ml)	1.8 (1.2-2.4)	2.0 (1.4-3.0)	3.1 (1.8-4.1)	<0.0001

Data are mean ± SD, frequency (percent), or median (interquartile range). HDL, high-density lipoprotein; LDL, low-density lipoprotein; GFR, glomerular filtration rate, UACR, urinary albumin-to-creatinine ratio. ^#^*p* values were calculated by one-way ANOVA test followed by the Tukey pairwise comparison.

**Table 3:** Associations between plasma FABP1 and FABP2 and diabetic nephropathy in fully adjusted multivariate models

Model adjusted for	Diabetic nephropathy		
	OR	95% CI	*p* value
Plasma FABP1			
Age, gender	1.01	1.00-1.02	0.009
Age, gender, BMI, SBP, DBP	1.01	1.00-1.02	0.017
Age, gender, BMI, SBP, DBP, fasting glucose	1.01	1.00-1.02	0.015
Age, gender, BMI, SBP, DBP, fasting glucose, lipid profile, smoking status	1.01	1.00-1.02	0.015
**Plasma FABP2**			
Age, gender	1.34	1.12-1.62	0.002
Age, gender, BMI, SBP, DBP	1.31	1.08-1.60	0.007
Age, gender, BMI, SBP, DBP, fasting glucose	1.31	1.08-1.60	0.007
Age, gender, BMI, SBP, DBP, fasting glucose, lipid profile, smoking status	1.30	1.05-1.60	0.014

Results of multivariate logistic regression analysis are presented as the OR of being in diabetic nephropathy status increases in plasma FABP1 and FABP2. BMI, body mass index; SBP, systolic blood pressure; DBP, diastolic blood pressure; FABP, fatty acid-binding protein; lipid profile: including total cholesterol, triglyceride, low- and high-density lipoprotein-cholesterol.

**Table 4:** Univariate and multivariate analyses of the impact of plasma FABP1 and FABP2 level on diabetic nephropathy

Factor	Tertiles of FABP1			
	T1 (95% CI)	T2 (95%CI)	T3 (95%CI)	*p* value
**All subjects**				
No. of cases/reference	15/74	38/51	41/49	<0.0001
Cut off FABP1 concentration (ng/mL)	<25.4	25.4-38.1	>38.1	
Univariate	1.00	3.68 (1.86-7.55)	4.13 (2.10-8.46)	<0.0001
Multivariate^a^	1.00	3.47 (1.59-7.88)	4.22 (1.85-10.03)	0.001
	**Tertiles of FABP2**			
**Factor**	**T1 (95% CI)**	**T2 (95%CI)**	**T3 (95%CI)**	***p* value**
**All subjects**				
No. of cases/reference	24/64	33/59	37/51	0.012
Cut off FABP2 concentration (ng/mL)	<1.52	1.52-2.32	>2.32	
Univariate	1.00	1.49 (0.79-2.83)	1.94 (1.03-3.67)	0.039
Multivariate^a^	1.00	1.34 (0.65-2.79)	1.62 (1.50-11.81)	0.041

Values shown are cut-offs of plasma FABP1 and FABP2 levels of all subjects, and odds ratios (ORs) with 95% confidence intervals (CIs). ^a^Adjusted for age, sex, body mass index, systolic blood pressure, diastolic blood pressure, fasting glucose, total cholesterol, triglycerides, high-density lipoprotein-cholesterol, low-density lipoprotein-cholesterol, and smoking. FABP, fatty acid-binding protein.

**Table 5:** Correlations among FABP1 and FABP2 and clinical and biochemical parameters in the enrolled patients

	FABP1		FABP2	
	r	*p* value	r	*p* value
Age	0.145	0.020	0.098	0.108
Gender	0.034	0.583	0.051	0.410
Body mass index	0.216	0.001	-0.012	0.849
Waist-to-hip ratio	0.202	0.001	0.013	0.827
SBP	0.074	0.231	0.121	0.047
DBP	0.054	0.386	0.065	0.291
Fasting glucose	-0.100	0.108	0.004	0.945
HbA1c	0.032	0.613	0.004	0.950
T-cholesterol	-0.026	0.679	0.126	0.039
Triglycerides	-0.004	0.954	0.053	0.385
HDL-cholesterol	-0.010	0.871	0.041	0.503
LDL-cholesterol	-0.024	0.705	0.100	0.102
Uric acid	0.226	0.0002	0.270	<0.0001
Creatinine	0.347	<0.0001	0.430	<0.0001
Estimated GFR	-0.337	<0.0001	-0.408	<0.0001
UACR	0.171	0.006	0.215	0.0004
Albumin	-0.195	0.002	-0.198	0.001
White blood cell count	0.070	0.261	0.098	0.109
Red blood cell	-0.192	0.002	-0.161	0.009
Hemoglobin	-0.124	0.045	-0.172	0.005
FABP1	-	-	0.206	0.001
FABP2	0.206	0.001	-	-
Fatty liver index	0.242	<0.0001	0.142	0.020
Current smoking	0.071	0.257	0.106	0.084

SBP, systolic blood pressure; DBP, diastolic blood pressure; HDL, high-density lipoprotein; LDL, low-density lipoprotein; GFR, glomerular filtration rate; UACR, urinary albumin-to-creatinine ratio; FABP, fatty acid-binding protein; TNF, tumor necrosis factor.
